# Cellular clarity: a logistic regression approach to identify root epidermal regulators of iron deficiency response

**DOI:** 10.1186/s12864-023-09714-6

**Published:** 2023-10-18

**Authors:** Selene R. Schmittling, DurreShahwar Muhammad, Samiul Haque, Terri A. Long, Cranos M. Williams

**Affiliations:** 1 Department of Electrical & Computer Engineering, North Carolina State University, Raleigh, USA; 2 Department of Biosciences, Rice University, Houston, USA; 3 Life Sciences Customer Advisory, SAS Institute Inc, Cary, USA; 4 Department of Plant & Microbial Biology, North Carolina State University, Raleigh, USA

**Keywords:** Iron deficiency, Epidermis, Arabidopsis, Logistic regression, Gene regulatory network, RNA-seq

## Abstract

**Background:**

Plants respond to stress through highly tuned regulatory networks. While prior works identified master regulators of iron deficiency responses in A. thaliana from whole-root data, identifying regulators that act at the cellular level is critical to a more comprehensive understanding of iron homeostasis. Within the root epidermis complex molecular mechanisms that facilitate iron reduction and uptake from the rhizosphere are known to be regulated by bHLH transcriptional regulators. However, many questions remain about the regulatory mechanisms that control these responses, and how they may integrate with developmental processes within the epidermis. Here, we use transcriptional profiling to gain insight into root epidermis-specific regulatory processes.

**Results:**

Set comparisons of differentially expressed genes (DEGs) between whole root and epidermis transcript measurements identified differences in magnitude and timing of organ-level vs. epidermis-specific responses. Utilizing a unique sampling method combined with a mutual information metric across time-lagged and non-time-lagged windows, we identified relationships between clusters of functionally relevant differentially expressed genes suggesting that developmental regulatory processes may act upstream of well-known Fe-specific responses. By integrating static data (DNA motif information) with time-series transcriptomic data and employing machine learning approaches, specifically logistic regression models with LASSO, we also identified putative motifs that served as crucial features for predicting differentially expressed genes. Twenty-eight transcription factors (TFs) known to bind to these motifs were not differentially expressed, indicating that these TFs may be regulated post-transcriptionally or post-translationally. Notably, many of these TFs also play a role in root development and general stress response.

**Conclusions:**

This work uncovered key differences in -Fe response identified using whole root data vs. cell-specific root epidermal data. Machine learning approaches combined with additional static data identified putative regulators of -Fe response that would not have been identified solely through transcriptomic profiles and reveal how developmental and general stress responses within the epidermis may act upstream of more specialized -Fe responses for Fe uptake.

**Supplementary Information:**

The online version contains supplementary material available at 10.1186/s12864-023-09714-6.

## Background

Iron (Fe) plays a critical role in essential physiological and biochemical pathways in plants [1–3]. Consequently, iron deficiency (-Fe) results in reduced plant growth and nutritional quality [4, 5], while excess iron can be lethal due to the accumulation of reactive oxygen species [6]. Plants are the primary conduit by which Fe and other essential minerals are mined from soil for animal uptake [7]. However, while Fe is the fourth most abundant element in soil, it is not easily utilized in its common form [8]. Thus, the challenge of engineering plants capable of maximizing iron uptake, while efficiently storing iron for consumption, plays an important role in addressing human and plant health issues.

Recent studies using the model plant *Arabidopsis thaliana* have led to the development of new and predictive mathematical models that describe regulatory processes involved in -Fe response in roots [9–14]. One of the most well-studied regulatory processes involved in -Fe response is Strategy I iron acquisition. During this response, the master regulator, basic helix-loop-helix (bHLH) transcription factor FER-LIKE FE DEFICIENCY INDUCED TRANSCRIPTION FACTOR (FIT) binds with bHLH heterodimers (bHLH100, bHLH101, bHLH38, and bHLH39), ETHYLENE INSENSITIVE3 (EIN3), and ETHYLENE INSENSITIVE3-LIKE1 (EIL1) [9, 14, 15] to transcriptionally activate the H + -ATPase (AHA2), which acidifies the rhizosphere and increases the solubility of Fe(III) near the root epidermis. Following acidification, FERRIC REDUCTASE OXIDASE2 (FRO2), reduces Fe(III) to Fe(II), and IRON REGULATED TRANSPORTER1 (IRT1), a membrane-localized metal ion transporter, transports iron across the plasma membrane [9, 16–20]. Upon iron uptake, genes such as *YSL-Like* and *FRD3*, which encode transporters of Fe chelates nicotianamine and citrate, respectively, are expressed to facilitate long-distance transport of Fe throughout the vasculature [21, 22].

High-throughput transcriptomic temporal analysis of *Arabidopsis* responses to iron deprivation has been critical in moving the field of iron homeostasis forward [9–11, 23, 24]. However, well-studied Strategy I iron acquisition transcriptional regulators (FIT, bHLH100/101, bHLH38/39) and their subsequent targets (AHA2, FRO2, IRT1) [9, 14–19, 25] are not completely described from these analyses, as molecular processes controlling Strategy I iron acquisition primarily occur in the outer cell layer of the root, the epidermis [9]. While every cell contains the same genetic information, distinct cell processes arise largely due to differences in gene regulation, thus stress response varies between cells and cell types [11, 24, 26–30]. For example, 48% of salt-responsive genes are regulated in the cortex under high salinity, while 28% and 31% are regulated in the stele and epidermis, respectively [11, 24, 26, 27]. POPEYE (PYE), another iron-responsive transcription factor, exhibits opposing cell-type specific regulation to mediate iron bioavailability [29]. Current iron-response networks typically represent gene regulatory relationships inferred from *Arabidopsis* whole root data and do not consider the cell-specific nature of regulation. In light of the critical role of the epidermis in Fe uptake, here, we focused on this cell type as a model for identifying cell-specific regulators of -Fe response.

While clustering transcriptional data generates groups of genes with common transcriptional profiles [31–33], inferring relationships between genes from different clusters remains challenging. Inter-cluster networks capture regulatory relationships where network nodes represent clusters of genes and edges represent relationships between clusters. Connections are typically inferred using cluster centroids or mean transcription profiles [10, 34]. Using cluster centroids may not be ideal as genes within the cluster may deviate from the mean cluster profile, potentially resulting in missed relationships or connections across clusters. A method that builds inter-cluster relationships based on individual transcriptional profiles might reveal crucial transcriptional responses, particularly in cases with significant intra-cluster variations of transcriptional profiles.

TFs in inter-cluster networks derived from stress response transcriptional profiles represent potentially important regulators that are differentially expressed. When working with time-course data with a control at each time point, differential expression is defined by the induction or repression of transcript abundance under the experimental conditions (e.g., iron deprivation) with respect to the control at that time point. Potential regulators that are not differentially regulated with respect to the control but have non-negligible transcriptional abundance would not be identified by this definition of differential expression, and thus would not be included in the set of genes used for downstream gene regulatory network inference. Transcription factors that fall into this category, however, may be differentially regulated outside of transcriptional mechanisms (e.g., post-transcriptional regulation), and still have the potential to differentially regulate genes that show up as differentially expressed. Prior work has shown that post-transcriptional regulation plays a key role in stress response [35–37]. Methods that integrate transcriptional profiles with other static data sets (e.g., DNA motif information [38–40]) have the potential to identify stress regulators that would otherwise be missed by traditional approaches.

In this work, we analyzed root epidermal transcriptomic data of plants exposed to -Fe for 36 h, taking samples at 6-h increments, and compared these profiles to existing whole root time course data. Set comparisons of differentially expressed genes allowed us to identify organ-level versus cell-specific responses and to parse out developmental genes from those induced or repressed by iron deficiency. We identified several TFs with no previously known role in -Fe response that were induced or repressed by -Fe. Utilizing a unique sampling method combined with a mutual information metric across time-lagged and non-time-lagged windows, we inferred a cluster-based GRN that identified relationships between clusters of functionally relevant differentially expressed genes. This network suggested that developmental regulatory processes may act upstream of well-known Fe-specific responses. By integrating time-series transcriptome profiles with static DNA motif information and interpretable machine learning models [41–43] (e.g., logistic regression with LASSO), we identified motifs that served as crucial features for predicting differentially expressed genes. Twenty-eight transcription factors (TFs) known to bind to these motifs were themselves not differentially expressed. This results in a putative list of TFs that may contribute to the differential expression of genes under -Fe but which are themselves potentially regulated outside of transcription and would otherwise be missed by traditional approaches. Overall, this work contributes to our overall understanding of -Fe response in epidermal cells and reveals how developmental and general stress responses within the epidermis may act upstream of more specialized -Fe responses for Fe uptake.

## Results

### Cell-specific experimental data uncovers genes associated with -Fe response in the epidermis

**Fig. 1:**
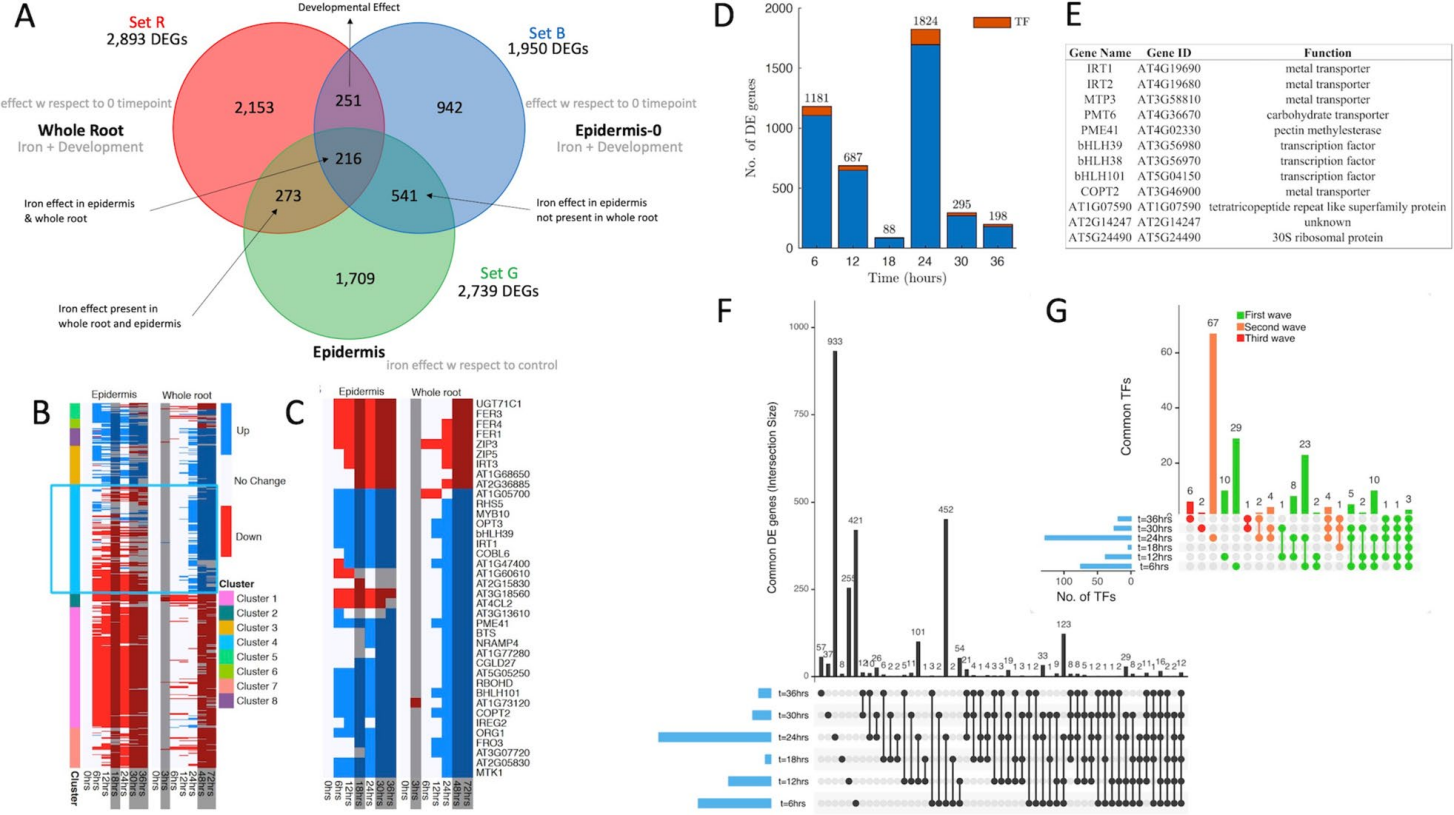
-Fe whole root and epidermis specific transcriptional comparative analysis. **A** DEG counts found in 1) whole root data using control at 0 time point only (Set Red), 2) cell-specific epidermis data using control at 0 time point only (Set Blue), and 3) cell-specific epidermis data using controls at all 7 time points (Set Green). The first two show differentially expressed genes involved in stress response & development. The first captures average behavior over several cell types and the second shows epidermis only. The third shows differentially expressed genes associated with iron response in the epidermis. **B** Heatmap comparing DEG expression in whole root and epidermis using control at 0 time point only. Blue box shows genes with significantly different expression patterns. Blue indicates up-regulation, Red indicates down-regulation. **C** Heatmap of expression for 38 known iron homeostasis DEGs in whole root and epidermis data using 0-time point control showing earlier activation in the epidermis vs. whole root. **D** DEG Gene counts at each time point identified using epidermis data using a control at each time point. The largest number of DEGs is seen at 24 h. The second largest is seen at 6 h. **E** List of genes that are differentially expressed at all 7 time points including 3 metal transporters and 3 known regulators of -Fe. **F** Upset diagram comparing sets of differentially expressed (under -Fe in the epidermis) genes at each time point. **G** Upset diagram showing the subsets of differentially expressed (under -Fe in the epidermis) transcription factors at each time point

Differences between genes expressed at the organ level and genes expressed specifically in the epidermis were first identified. Microarray data capturing expression activity at 7 time points (0, 3, 6, 12, 24, 48, and 72 h) and previously analyzed relative to the control sample at the 0-h time point were used to capture root (organ)-level gene expression activity [24]. Epidermis transcriptome data were obtained by sequencing marker-assisted fluorescence-activated cell sorted samples targeting root epidermal cells under -Fe and + Fe (control) at 7 time points (0, 3, 6, 12, 18, 24, and 36 h) (see Methods). These two time-course datasets were compared based on three sets of differentially expressed genes: Set R[ed], which was composed of genes differentially expressed in the microarray data at any time point with respect to the 0-h + Fe (control) time point (Fig. 1A Set R[ed]); Set B[lue], which was composed of genes differentially expressed in the epidermis RNA-Seq data at any time point with respect to the 0-h + Fe (control) time point (Fig. 1A Set B[lue]); and Set G[reen], which was composed of genes differentially expressed in epidermis RNA-Seq data at any time point with respect to the + Fe control at that time point (Fig. 1A Set G[reen]).

Set R (organ-level) contained 2,893 DEGs (Supplementary Table S0^1^), while Set B (cell-specific) which also used a control at the 0-h time point only, uncovered 1,950 DEGs (Fig. 1A Set B). A comparison of these two sets, which both used a 0-h control only, identified differences that were not associated with having a control at all time points. We identified 2,739 DEGs (Set G) in the epidermis using + Fe controls at all time points (Supplementary Table S0^2^). Figure 1A compares the numbers of genes in each set. Comparing whole root and epidermal DEGs identified using only the 0-h + Fe control revealed 467 common genes (intersection of red and blue sets in Fig. 1A), suggesting we missed approximately 50% of the epidermis-specific iron response DEGs when solely using whole root data.

### Control samples at every time point identify iron response genes in the epidermis

Differential expression with respect to only the 0-h time point (control) identifies genes that change significantly at the time stress is applied, resulting in a set of genes that change as a result of stress and genes that change as a result of development. Differential expression analysis with respect to a control at every time point ensures that DEGs are associated with stress response, rather than development. We isolated the iron response from developmental effects by comparing the epidermis-specific DEGs identified using a 0-h + Fe control only (Set B) to those identified using an + Fe control at every time point (Set G; Fig. 1A).

We identified 1,709 iron-responsive genes that would have been missed had we identified DEGs solely with respect to the 0-h time point control using the whole root or epidermis data (Fig. 1A). While microarray and RNA-Seq both measure transcript expression, microarray results are limited to genes with representative probes on the array, and produce relative measurements based on fluorescent activity [33, 44, 45]. RNA-Seq, on the other hand, identifies all transcripts in the sample within a detectable range using a reference genome and produces absolute transcript counts [45]. RNA-Seq’s ability to identify a broader group of genes with increased sensitivity means it can identify and quantify more genes than microarray data. This might account for the differences we observed with iron-responsive genes between the microarray and RNA-Seq datasets. However, given the comparable number of genes identified using microarray or RNA-Seq, it seems likely that this effect is negligible.

### Epidermal transcriptomic expression patterns reveal expression that differs from whole root patterns

A comparison of the profiles of the 467 genes that were differentially expressed in both the whole root and the epidermis transcript data (Fig. 1A) uncovered several differences (Figs. 1B and C). Figure 1B reveals differences across all 467 genes as a heatmap of clustered expression patterns for the epidermis vs. the whole root. Eight clusters were identified using hierarchical clustering of expression patterns from whole root and epidermis (Fig. 1B). Clusters 1, 3, 5, 6, 7, and 8 showed delayed expression in the whole root (48 h) compared to the epidermis (6 h) and in some cases, reversed directionality (i.e., activated vs. repressed and vice versa). Cluster 4 showed a delayed activation pattern (12 and 24 h vs. 6 and 12 h in the epidermis) and an almost complete change in directionality: up-regulation in the whole root vs. down-regulation in the epidermis.

Thirty-eight known iron homeostasis genes exhibited earlier activation or repression in the epidermis, compared to their expression in the whole roots (Fig. 1C, 6 hours vs. 12 and 24 h in the whole root). IRT1, regulated by FIT and considered the main iron transporter involved in iron uptake from the rhizosphere, [9, 16, 25] and MYB10, an -Fe-induced transcription factor [46], were upregulated in the epidermis at 6 h, whereas they did not appear upregulated in the whole root until 24 h. Two FIT interactors, bHLH39 and bHLH100, as well as OPT3 and COPT2, iron and copper transporters, respectively, were upregulated after 12 h of exposure to -Fe in the epidermis, but at 24 h in the whole root dataset. FER1 and FER4, genes that bind iron to prevent oxidative stress [47], were repressed in the epidermis as early as 6 h but repressed at 24 h of -Fe in the whole root dataset. Earlier induction or repression of known iron homeostasis genes in the epidermis compared to that in the whole root suggests that early epidermal activity is masked by collective activity in the whole root. These findings are supported by previous studies that characterized cell-type specific transcriptional changes under salt and -Fe stress [11, 24, 26].

### Epidermal expression patterns reveal response waves

We analyzed the 2,739 DEGs identified using the epidermis data and a control at every time point (Set G) to assess overall epidermal expression dynamics. We found the most DEGs and differentially expressed TFs at 6 (1,181 DEGs) and 24 h (1,824 DEGs) (Fig. 1D), while the 18-h time point resulted in the lowest number of transcriptional profile changes (88 DEGs) and the fewest TFs (4) (Fig. 1D). This suggests that these time points represent the initiation of transcriptional changes that induce a cascade of downstream responses. Twelve genes, listed in Fig. 1E, were differentially expressed at all sample time points, including four metal transporters and three known -Fe regulators. STOP2, previously identified as regulating several genes for aluminum stress and low pH tolerance, was differentially expressed in our data at 5 out of 6 time points [48]. Tsai and Schmidt (2020), investigating transcriptomic changes under optimal and high pH under -Fe, found that STOP2 was moderately induced under optimal pH, but highly repressed under high pH in iron-deficient conditions [49]. Since -Fe triggers the activity of epidermal proton pumps to acidify the rhizosphere and increase iron solubility [19], STOP2 likely plays an indirect role in iron response by regulating prevailing pH conditions. While STOP2 was previously identified as a minor isoform of STOP1, which controls pH and Al response [48], we did not find STOP1 to be substantially regulated in our datasets. Thus, STOP2 may play a more prominent role in controlling -Fe-induced pH changes at the rhizosphere-epidermal interface.

To characterize gene activity, we described each gene’s activity by the time points at which they were differentially expressed (Fig. 1F). Focusing on activity patterns of TFs (Fig. 1G), we identified three waves of transcriptional responses: wave 1 (activity before 12 h) contained the largest number of TFs (94), wave 2 (activity between 12 and 18 h) included 78 TFs and wave 3 (activity only after 30 h), 9 TFs. This activity suggests an organized cascade of transcriptional stress response which is discrete from wave to wave.

### Expression analysis and inter-cluster GRN reveal epidermis-specific regulators and response genes

Using the Dirichlet process Gaussian process mixture model (DPGP) clustering algorithm [50], we generated 50 co-expression clusters using the max normalized gene expression of our 2,739 DEGs (Supplementary Table S0^2^). Two clusters, 25 and 26, were enriched for iron-related Gene Ontology (GO) terms (adjusted p-value of 0.05). Most genes and TFs in these two clusters were previously known iron response genes. We did not find enriched GO terms in other clusters, probably due to smaller cluster sizes. However, we identified multiple cluster-specific TFs within the smaller clusters with known regulation adjacent or related to -Fe roles in development and stress response. Clusters 36 and 37 contained GL2, TRY, RHD6, and LRL1, TFs related to root hair development, while Cluster 1 contained several TFs involved in ABA response. Expression patterns for the 50 clusters are provided in Supplemental Figure S1.

**Fig. 2:**
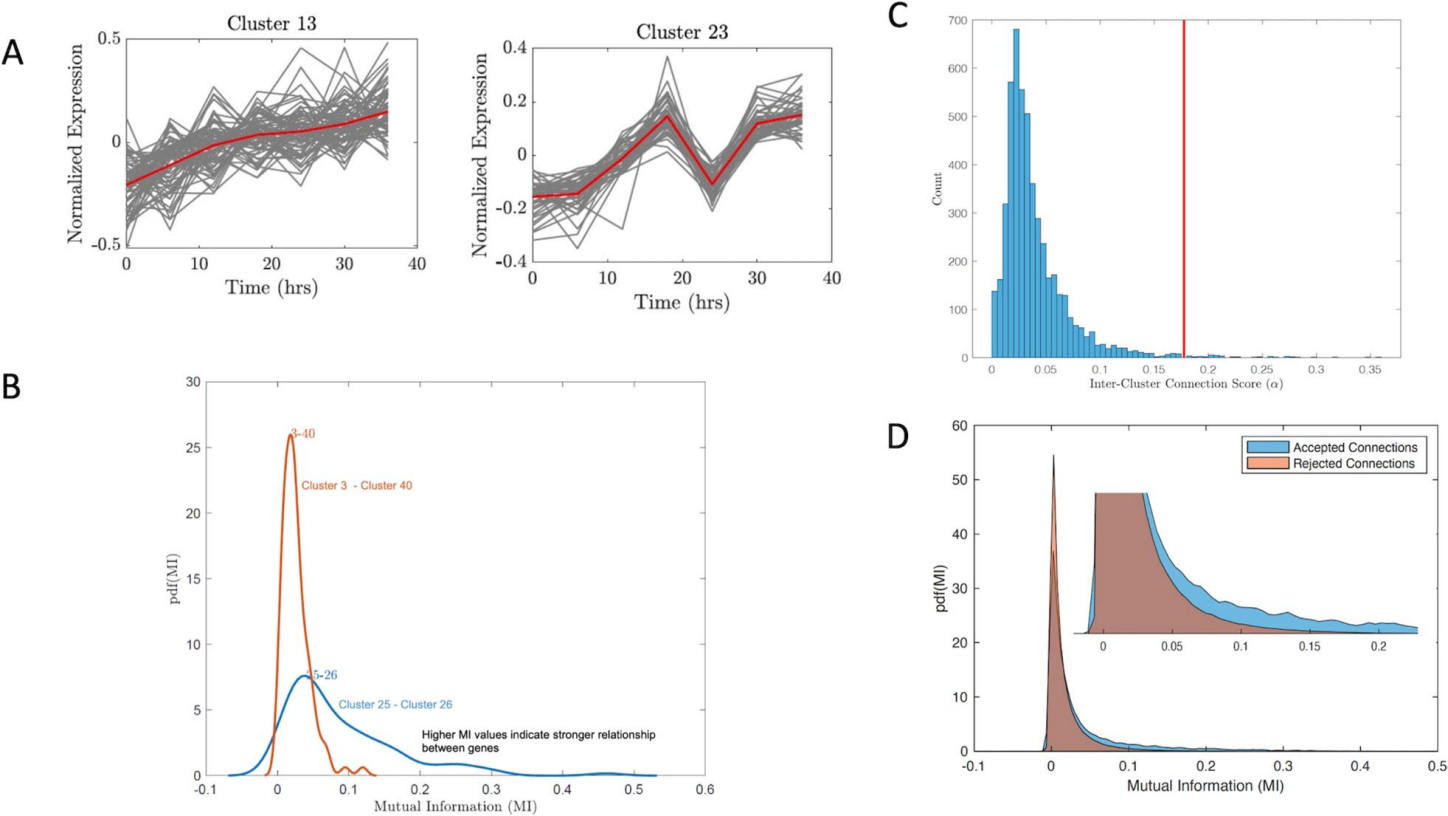
Computationally efficient mutual information sampling technique to infer gene regulatory network. **A** These examples show cases in which the mean is not a good representation of individual gene expression, **B** Example distribution of inter-cluster MI scores for 2 clusters, **C** Distribution of alpha values for all clusters **D**) MI scores for accepted vs. rejected connections for all clusters

To identify new functional relationships, we inferred and examined inter-cluster relationships. Previous studies and our results indicate that genes with similar biological functions show similar expression patterns and are more likely to belong to the same cluster (intra-cluster relationships). Likewise, it is logical to assume clusters containing regulator genes should have some causal relationship to clusters containing target genes (inter-cluster relationships). Previous works that developed cluster networks used cluster means or centroids to quantify cluster correlation [34, 51]. However, the range of transcriptional profiles within a cluster (intra-cluster variation) may result in the cluster centroid being a poor representation of all genes within the cluster (Fig. 2A) and result in the inability to infer informative causal inter-cluster relationships. To address this, we implemented a sampling-based scheme that measured the strength of a cluster relationship based on the distribution of Mutual Information (MI) [52] between individual genes belonging to clusters. This allowed us to incorporate gene-level expression patterns while maintaining computational efficiency. MI is a well-known metric for quantifying causality between pairs of genes [53].

Using our sampling technique for each inter-cluster connection, we generated probability distribution functions of MI scores (Fig. 2B). High MI scores indicate strong pairwise relationships between genes, which we expected to find in the tails of these distributions (higher MI values). We ranked all cluster connections using no time lag and a 6-h time lag to catch connections that would be missed using only a 0-time lag. We defined α_ij_ as the 90th percentile value of the MI score distribution calculated for genes in the *i*th and *j*th clusters. Larger values of α indicated MI distributions with stronger connections in the top 10% of their MI values while smaller values of α indicated MI distributions with weaker connections in the top 10% of their MI values. We generated the distribution of α_ij_ for all *ij* cluster pairs. We then identified a natural separation in this distribution (Fig. 2C) from which we could distinguish MI distributions with strong connections from distributions with weak connections. We identified a threshold of *ɑ* = 0.1775 as shown in Fig. 2C, which was the center value of an empty bin that created a natural separation between lower and higher values of ɑ. Examination of the distribution of Mutual Information values for connections that were accepted vs. rejected using this threshold of α (Fig. 2D) shows that while the mode of their distributions is similar, the accepted cluster connections have a higher density in the tails.

**Fig. 3:**
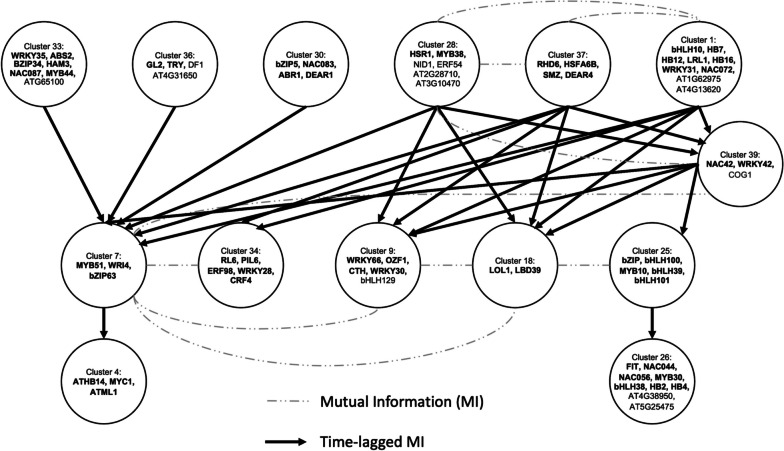
Inter-cluster GRN. Inter-cluster GRN generated using DPGP clustering coupled with sampling-based scheme to measure the strength of a cluster relationship. Important TFs for each cluster are labeled. Dash lines indicate MI between gene pairs and solid arrows represent time-lagged MI

We identified 33 potential interactions among 14 clusters (Fig. 3) from the accepted connections. Connections uncovered between FIT and binding partners, bHLH100, bHLH101, and bHLH39 have been confirmed experimentally in prior works [14, 54]. We used known TFs in the clusters to categorize biological functionality using existing literature and the TAIR database [55]. Our network included connections between Cluster 1, which contains FER1; Cluster 28, which contains FER3 and 4; and Cluster 39, which contains NAC042 (JUB1) and AT3G20340. Sudre et al. (2013) found 54 genes, including NAC042 and AT3G20340, were significantly affected under 3 conditions: *atfer1-3–4* mutant vs. WT both under iron sufficiency, *atfer1-3–4* under excess iron vs. WT, and *atfer1-3–4* vs. WT both under excess iron [56]. These findings support our network’s biological relevance and provide future avenues of investigation on -Fe response in epidermal cells. Potential novel -Fe responsive genes identified also have other biological functions, e.g., root hair development (GL2, TRT, RHD6), ABA response (WRKY31, CTH/AtTZF1), and nitrate/phosphate response (WRKY42, HRS1, NAC42). Two clusters containing TFs associated with root hair development, 1 and 37, are connected to Cluster 39 which contains nitrate/phosphate response TFs. Liu et al. (2020) reviewed nitrate regulation associated with lateral root and root hair development and reported that both are gradually inhibited in a homogeneous nitrate environment [57]. These relationships suggest that iron deprivation triggers developmental and more general abiotic stress response within the epidermis that then induces downstream specialized processes for iron uptake activities.

### Logistic regression models identify TFs associated with motifs important for predicting differential expression in epidermal data

**Fig. 4:**
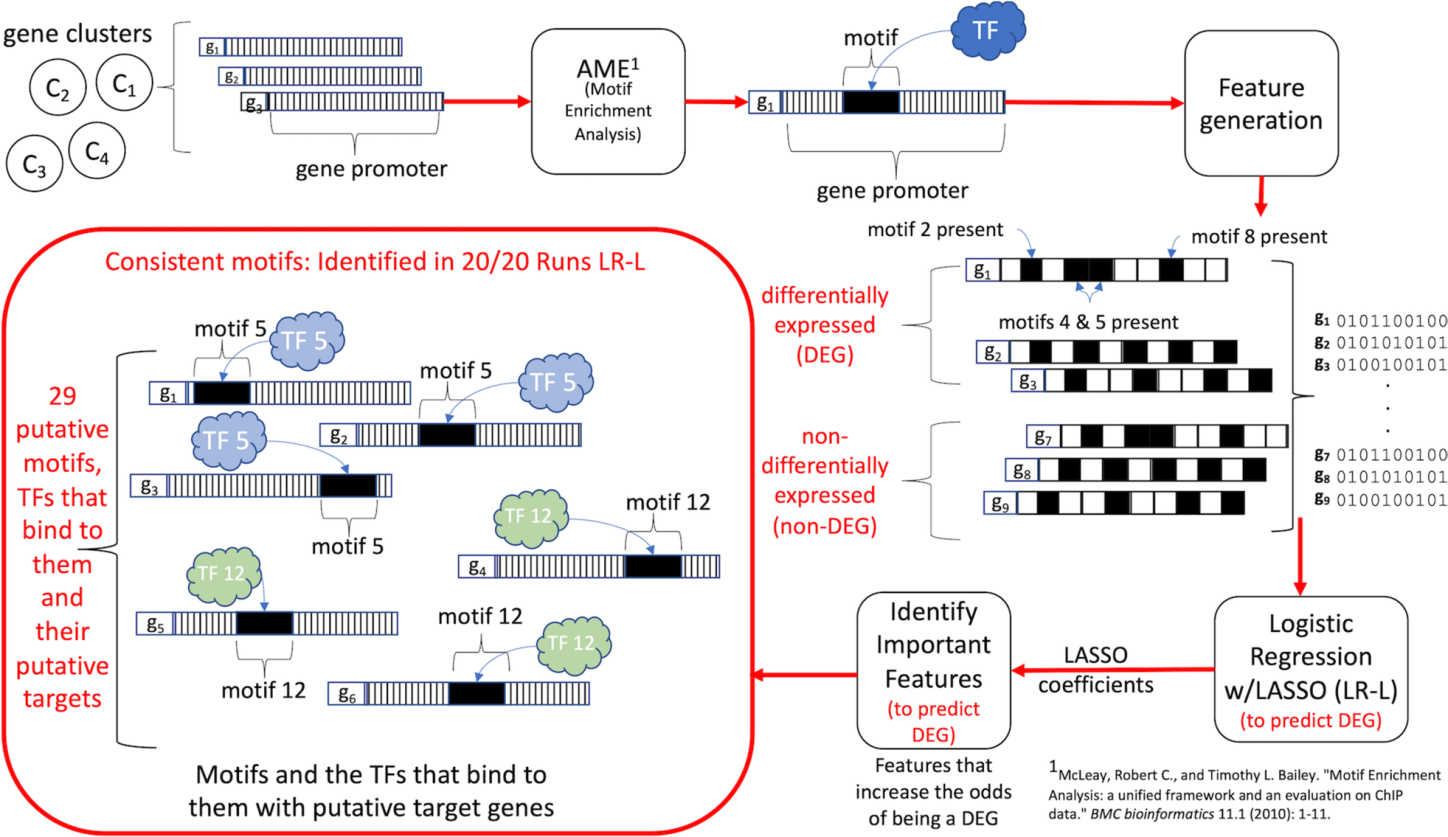
Machine learning pipeline to identify motifs/TFs important for predicting differential expression

In the previous section regulators of iron epidermal deficiency response were identified from a pool of differentially expressed genes. By integrating static data (DNA motif information) and our time-series transcriptomic data into interpretable machine-learning models, we uncovered regulators that influence genes that were differentially expressed under -Fe, but which were not differentially expressed themselves. In our pipeline (Fig. 4), we first identified known cis-elements enriched in the promoter region 1000 bp upstream of the transcription start site as in Schwarz et al. (2020) using genes in the clusters previously generated using the DPGP algorithm [50, 58]. We removed cluster 50 from further analysis since no motifs were identified for that cluster.

We found 337 motifs enriched in our gene clusters (Supplementary Table S0^3^) and generated features using the presence (0) or absence (1) of a motif in the promoter of each gene (see Methods). These features were input into a logistic regression (classification) model to identify motifs important for predicting differential expression. Given known class labels (e.g., differentially expressed, 1, and non-differentially expressed, 0), classification models can identify predictors that are associated with a response [58–62]. While previous works have successfully used Random Forest and Support Vector Machines, logistic regression models with LASSO regularization provide highly interpretable results in addition to feature selection for this task [41–43, 63].

We hypothesized that motifs that increased the likelihood of a gene being differentially expressed would be associated with stress response. We used the model coefficients to identify important motifs. In logistic regression, coefficients represent how a predictor increases or decreases the log odds of the response relative to the positive (i.e., response = 1 or “differentially expressed”) class. Since coefficients are associated with changes in the log odds, we chose motifs for which ${e}^{x}>1,$ where *x* is the coefficient [64]. Due to stochasticity introduced by LASSO regularization, we ran the algorithm 20 times and identified motifs that were consistent predictors across all runs. This uncovered 29 consistent motifs of interest (MOI; Supplemental Table S0^4^). By generating a model that included all differentially expressed genes, as opposed to one model per cluster, we were able to identify MOIs that were influential over multiple clusters. We extracted TFs known to bind to the 29 consistent MOIs (putative regulators, provided in Supplemental Table S0^4^) and the set of genes with the MOI in their promoter (putative targets, provided in Supplemental Table S0^5^) using the motif-finding tool, AME [65], which identifies biologically verified TFs using the DAP-seq database [66].

### Predictive models identify TFs that may be post-transcriptionally regulated

To better characterize the function of the identified TFs, we explored their expression patterns and found that only one TF, ANAC070 (AT4G10350), was differentially expressed with respect to the control (+ Fe) at any time point. We performed GO analysis on these putative regulators to determine their biological relevance and found that 6 putative regulators were associated with terms specific to root development, one with the epidermis, and two with hormone pathways (Supplemental Table S0^4^). All of these activities have been associated with iron stress response and root hair development, which is affected by -Fe [67, 68]. Next, we determined the extent to which putative targets of these TFs included known iron homeostasis genes. Thirteen of our 29 putative regulators had at least one known iron homeostasis gene in their putative target sets. Putative targets included known iron stress-responsive genes: BRUTUS(BTS), IRT1, and FRO2, as well as regulators (TFs) of the iron stress response including FIT, bHLH100, and bHLH101 (Supplemental Table S0^5^).

To assess the gene function of the putative targets for each of our TFs, we performed GO enrichment analysis and identified 876 unique, statistically significant (adjusted *p*-value < 0.05) terms across all putative regulators. Supplemental Figure S0^2^ shows a subset of terms identified. A complete list of TFs, their GO terms, and FDR are included in supplemental data (Supplemental Table S0^6^). The putative targets of six of our 29 consistent TFs produced no statistically significant terms.

Since changes in root hair development are a hallmark phenotype of iron deprivation and these changes occur in the epidermis [11, 24], we focused on statistically significant terms from the GO analysis that included “iron”, “epidermis” and “root”. Targets for eleven of our TFs had terms associated with iron response and homeostasis, while 9 had terms associated with root morphogenesis, development, or regeneration, and 5 had terms associated with epidermis development and cell fate (Supplemental Tables S0^7^, S0^8^, and S0^9^). This suggests the MOIs/TFs we identified could be involved in iron response in the epidermis. Two of the MOIs/TFs with “root” in their GO terms and one with “epidermis” had no known iron homeostasis genes in their targets and could represent previously unidentified iron response genes.

### Non-differentially expressed regulators are associated with iron-related pathways and activity

Many of the putative regulators we identified were expressed at some level (Supplemental Figure S0^3^) and several have -Fe RPKM measurements with similar magnitude to bHLH101, a FIT binding partner that is differentially expressed under -Fe compared to + Fe control at every time point (Supplemental Figure S0^3^). Thus, we suspect that these non-differentially expressed transcription factors represent high-level regulators that themselves are regulated post-transcriptionally, not unlike several other -Fe-responsive TFs [69, 70]. A review of the literature indicates that post-transcriptional regulation is associated with four of our non-differentially expressed putative regulators, including the DOF family of transcription factors [71]. CUC1 was found to be targeted by miR164, which provides evidence of post-transcriptional regulation [72], and Huang et al. (2018) determined that PIF7 activity is regulated by phosphorylation in shade-induced nuclear localization [73], while LHY1 has also been found to be post-transcriptionally regulated [74–77].

**Fig. 5:**
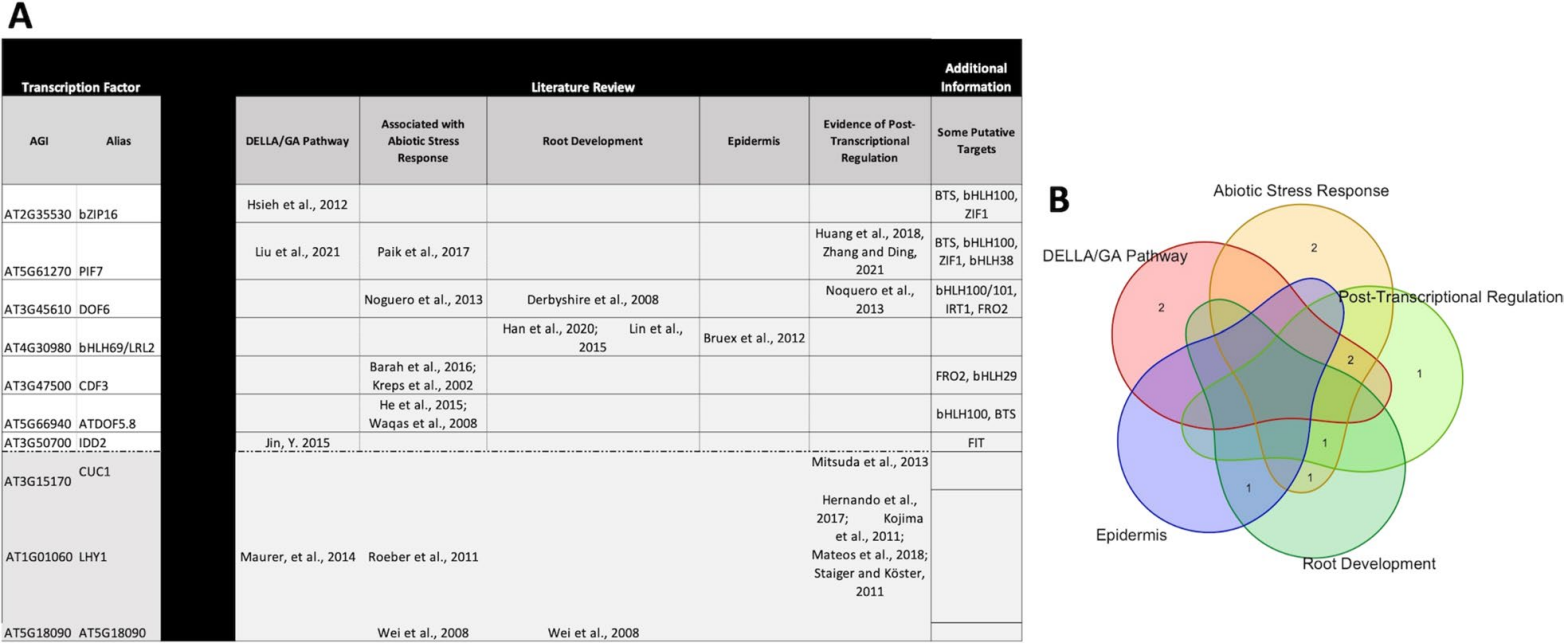
Subset of putative regulators identified using logistic regression with LASSO. **A** Putative TFs investigated in literature review. Subset included 13 TFs with known iron response genes in their putative targets and three additional TFs. References were identified for the topics listed in the table for 10 of the 16 TFs investigated. **B**) Venn diagram showing overlap between topics covered in the literature review for TFs listed in **A**

Post-transcriptional regulation is critical in abiotic stress response [35, 37, 78]. To explore whether the regulators we identified might be involved in iron deficiency response in the epidermis, we performed an extensive literature review. We focused on the subset of 13 putative regulators with known iron response genes in their targets and included three additional TFs with no known iron genes in their targets (CUC1, LHY1, and AT5G18090) to explore how these putative regulators and their targets might be associated with -Fe. Ten of the 16 TFs we reviewed were associated with one or more activities implicated in -Fe response (Fig. 5A and B) [71–77, 79–94]. These TFs represent a small testable set of previously unidentified iron stress response regulators.

Six of the TFs are implicated with abiotic stress response including, PIF7 [90], DOF6 [71], CDF3 [79, 86], ATDOF5.8 [83, 91], LHY1 [88] and AT5G18090 [92]. Putative targets associated with some of these TFs include BTS, bHLH100/101, bHLH38, bHLH29, IRT1, FRO2, and ZIF1 (Fig. 5A). While a universal stress response pathway could explain this, research by Iyer-Pascuzzi et al. (2011) did not find evidence of one [27]. However, they and others have found that regulators may respond to multiple stresses [24, 27, 86].

Four TFs are associated with the DELLA/gibberellin pathway, which has been implicated in FIT regulation [68]: bZIP16 [84], PIF7 [89], IDD2 [85], and LHY1 [88]. Putative targets of these four TFs include BTS, FIT, bHLH100, bHLH38, and ZIF1. LHY1 has not previously been identified as an iron stress response regulator. However, Maurer et al. (2014) found that LHY1 is more highly expressed under -Fe in a triple mutant background (*bhlh39-1 bhlh100-1 bhlh101-1*) [88]. Given that changes in LHY1 are associated with mutants of its putative targets a feedback mechanism may be involved.

Three TFs are associated with root expression or development: DOF6 [81], bHLH69/LRL2 [80], and AT5G18090 [92]. Root development and changes in root hair length are known phenotypes of -Fe [11, 95, 96]. Putative DOF6 targets include bHLH100/101, IRT1 and FRO2. DOF6 is associated with root radial pattern formation according to TAIR and is expressed in all three developmental zones (meristematic, elongation, and differentiation), but is pericycle-specific [81]. bHLH69 is involved in root hair formation and root epidermis development, and mutants show root hair defects [80]. AT5G18090 is induced in response to manganese deficiency, which is associated with stimulated root hair elongation [92]. Notably, there is evidence showing iron and manganese crosstalk via IRT1, an iron transporter that nonspecifically transports manganese under -Fe [25], increasing its levels in roots [11, 29].

Only one of our TFs, IDD2, is associated with epidermal root hair differentiation [85]. However, the relationship between this TF and the DELLA/gibberellin pathway makes it a prime TF for further investigation to determine its role in the -Fe response. While biological confirmation is required, this work uncovered connections between non-differentially expressed regulators and mechanisms associated with iron stress response and provides several avenues of future inquiry.

## Discussion

Gene set comparisons between organ- and cell-level data using a 0-h + Fe control sample identified 1,483 genes associated with epidermal activity that would not have been found without cell-specific measurements (Fig. 1A). By including a + Fe control sample at every time point, an additional 1,709 genes were identified that would have been missed if only a 0-h control were used (Fig. 1A). These results highlight the need for cell-level data with controls at every time point, as well as the benefit of gene set comparisons across datasets. While a complete comparison between whole root and epidermis data was not feasible since microarray data does not contain the whole transcriptome, our comparative transcriptional analysis shows evidence of early activation in the epidermis in response to -Fe along with complete transcriptional changes between the whole root (up-regulated) and the epidermis (down-regulated; Fig. 1B and C).

The cluster GRN (Fig. 3), developed using gene-level comparisons in a computationally efficient manner, identified relationships between transcription factors that mirror those found using whole root data, while also revealing novel putative regulators that act upstream of known -Fe response genes. In many cases, these early response regulators play a role in development and stress responses. A logistic regression with LASSO modeling approach uncovered regulators that may act upstream of known -Fe response processes, yet are not transcriptionally regulated by -Fe. Plants rely on a highly tuned regulatory system for stress response due to their sessile nature. Regulators revealed by our approach, such as CUC1, LHY1, and IDD2 may be post-transcriptionally controlled to fine-tune responses and enable quick transitions in response to Fe deprivation within the epidermis [36]. Notably, many of these putative upstream regulators also play a role in development and general stress response [85, 88, 97].

We propose a potential connection between the high-level putative regulators we identified, which are associated with development and general stress response, and -Fe response in the epidermis. The root epidermis is highly sensitive to stress and readily exhibits developmental, physiological, and biochemical alterations in response to nutrient availability [93, 94]. One of the most well-studied responses to -Fe stress is the induction of the Strategy I response within the root epidermis. This response relies upon membrane localization and induction of the critical FRO2, IRT1, and AHA2 protein complex that induces rhizosphere acidification, ferric reduction, and Fe uptake into epidermal cells [98].

**Fig. 6:**
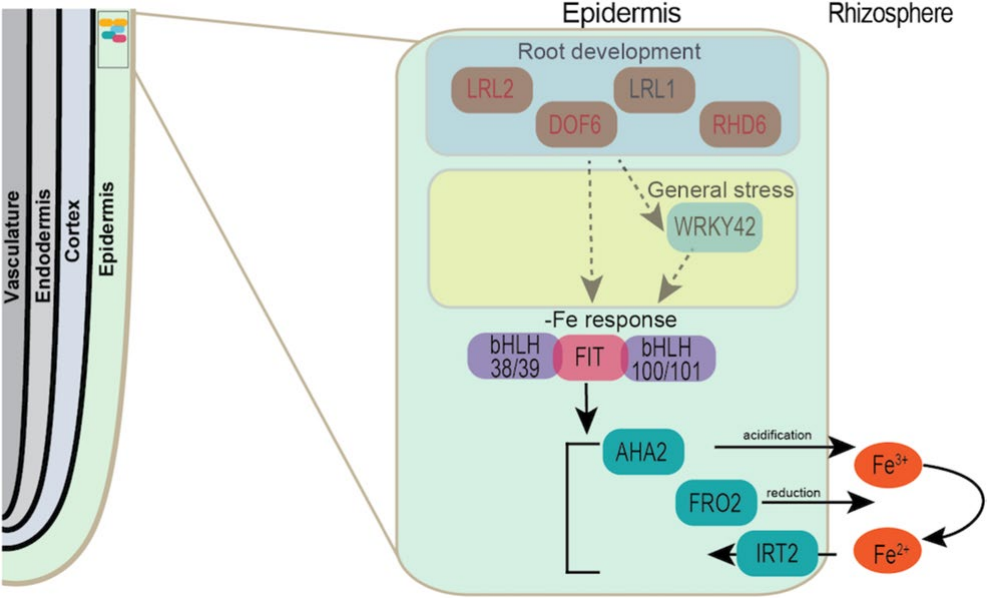
Hypothesized root epidermal -Fe mechanism. Model depicting epidermal root development (blue box) and general stress responses (yellow box) epistatic to the known -Fe response. Dash grey arrows indicate direct or indirect connections identified by the DPGP clustering and Logistic regression with LASSO modeling algorithms on the -Fe spatiotemporal RNA-seq data. Solid black arrows indicate confirmed direct -Fe interactions. Genes in red are potentially post-transcriptionally regulated

Our analysis revealed that several known developmental regulators, such as DOF6, bHLH69/LRL2, WRKY42, and RHD6, might directly or indirectly target FRO2 and IRT1, as well as their bHLH regulator, FIT, and FIT’s binding partners, bHLH100 and bHLH101, as well as other Fe homeostasis genes. Taken together our spatiotemporal RNA-seq and modeling of epidermal-specific transcriptional responses reveal a mechanism whereby developmental and general stress responses are epistatic or parallel to the early -Fe stress response within the root epidermis, which then later induce more specialized -Fe responses for Fe uptake (Fig. 6). Figure 6 hypothesizes connections between transcription factors associated with development, general stress response, and -Fe response.

## Conclusions

This work uncovered key differences in -Fe response identified using whole root data vs. cell-specific root epidermal data, and using a 0-h control vs. controls at every time point. Machine learning approaches, which employed interpretable models combined with additional static data, identified potential high-level regulators of -Fe response that would not have been identified solely through transcriptomic profiles. These regulators reveal how developmental and general stress responses within the epidermis may act upstream of more specialized -Fe responses for Fe uptake.

## Methods

The *Arabidopsis* pWER::GFP epidermis marker line (WEREWOLF; AT5G14750: Lee & Schiefelbein, 1999) in Columbia (Col-0) accession was used to perform all experiments [99]. Seeds were surface sterilized using 70% ethanol for 2 min followed by 30% bleach and 0.02% Triton X-100 solution for 15 min. Seeds were rinsed three times with sterile water and stratified at 4 °C for 2 to 3 d before being plated on nylon mesh (Genesee Scientific Cat 57–103) on top of solidified media to facilitate transfer. Seeds were grown in a vertical position in a Percival incubator with 16 h of daily illumination and 8 h of dark at 22 °C. Iron-sufficient (+ Fe) and deficient media is standard Murashige and Skoog media (Caisson Labs Cat MSP33-50LT) with 0.05% MES, 1% sucrose, 1% agar, and 0.1 mM FeEDTA. Ferrozine (300 μM), an iron chelator, was added to make deficient media.

Seedlings were shifted from iron-sufficient media to sufficient and deficient media, on the 7^th^ day of growth, equating to 0-h. After 7d transfer, an additional 6 h, 12 h, 18 h, 24 h, 30 h, and 36 h of growth occurred. After which, roots were harvested for protoplasting followed by fluorescence-activated cell sorting [100]. Three replicates of the epidermis samples were collected and analyzed.

RNA was extracted using RNAeasy Plant RNA Purification Kit (Qiagen Cat. 74,904). cDNA synthesis and amplification were performed using the SMARTer Low Input RNA Kit (TaKaRa Cat. 634,940) for sequencing. North Carolina State University Genomic Science Library facilities sequenced the library using an Illumina HiSeq 2500 sequencing machine, with 125 bp single-end reads.

Adapter contamination and low-quality reads were assessed using fastQC [101]. Adapter contents were trimmed, and low-quality reads discarded using fastq-mcf [102]. Clean reads were mapped to The Arabidopsis Information Resource (TAIR)10 reference genome [55] using tophat2 [103–105]. More than 92% of reads were mapped to the reference genome for all the samples. From the mapped reads we obtained the read count (RPKM) for each gene using the RSubread package [106].

Differentially expressed genes (DEGs) were identified using edgeR [107] package, using glmQLFtest for DE analysis. DE genes were identified with respect to the 0-time point or with respect to the control using adjusted p-values [108]. A maximum false discovery rate (FDR) of 0.05 and minimum log fold change threshold of 0.75 was applied for identifying differentially expressed genes.

To cluster gene expression trajectories, we used a Dirichlet Process Gaussian Processed (DPGP)-based algorithm [50]. DPGP can capture time course dependencies and is suitable for modeling time series data. Data was max normalized prior to clustering using the following equation

$${N}_{t}^{i}=\frac{{R}_{t}^{i}-\frac{{\sum }_{k=1}^{t}{R}_{k}^{i}}{T}}{{R}_{k}^{i}}$$

Where ${N}_{t}^{i}$ is normalized expression of gene *i* at sampling time point *t*. T is the total number of time samples, and ${R}_{k}^{i}$ is the RPKM gene expression value of gene *i* at time *t*.

Latin Hypercube Sampling (LHS) [109] was used to randomly sample TFs from each DPGP-generated cluster (with repetition). We calculated 0- and 6-h time lagged mutual information (MI) scores between pairs of randomly sampled TFs from different clusters using [53]. Mutual information between the expression patterns of two genes (random variables) (G1,G2) is defined as

$$I({G}_{1};{G}_{2})={\iint }f({G}_{1}, {G}_{2})loglog \left(\frac{f({G}_{1})f({G}_{2})}{f({G}_{1},{G}_{2})}\right)d{G}_{1}d{G}_{2}$$

Where $f({G}_{1},{G}_{2})$ is the joint distribution, and $f({G}_{1})$, $f({G}_{2})$ are marginal distributions of the random variables representing the gene expression patterns. We generated a distribution from the MI scores calculated for each pair of clusters. We ranked all possible cluster connections based on the 90th percentile (near the tail end) of the MI value and called this score ɑ. We calculated ɑ for all possible cluster connections, generated a distribution of ɑ and identified an appropriate threshold which was applied to identify accepted connections between clusters.

Promoter regions 1000 bp upstream [58] of the transcription start site for genes in each of the 50 DPGP clusters were identified. We then used the Analysis of Motif Enrichment (AME) algorithm [65] using default settings and the DAP-seq database [66] to identify enriched motifs. AME provided a list of motifs, TFs that bind to them, and the genes that contained the motif in their promoter (putative targets). We generated a binary feature for each gene by encoding presence (1) or absence (0) of the motif for each gene. We encoded an equal number of non-differentially expressed genes (absolute log2FC for all time points < 0.251). We chose a more restrictive threshold than Schwarz et al. (2020) (0.4), to provide us with enough negative samples while ensuring that expression was negligible [58]. We set class labels for differentially expressed to 1, and non-differentially expressed to 0. These features and labels were used as input to Logistic Regression models with LASSO.

We performed logistic regression with LASSO using the R package glmnet. Important features were identified by using model coefficients for which (exp(*x*)) was > 1 where *x* was the model coefficient [64]. Since the use of LASSO can result in different coefficients from run to run, we generated 20 models and focused on motifs identified in all 20 runs. This allowed us to capture the variability of the algorithm and maintain the exploratory nature of the task.

GO terms associated with TFs that are known to bind to our motifs were identified using the TAIR database [55]. GO enrichment of putative targets was performed using the R library topGO [110].

### Supplementary Information

**Additional file 1:** **Figure. S01.** Expression profiles of 2,739 DEGs. 50 clusters were identified using DPGP clustering algorithm. Cluster 25 and 26 were enriched in iron related GO terms. **Figure. S02 **GO Enrichment of putative targets for a subset of GO terms. **Figure. S03.** Expression values of 29 TFs identified using Logistic Regression.

**Additional file 2: Table 01.** List of 2,893 differentially expressed genes identified in whole root data. **Table 02.** List of 2,739 differentially expressed genes identified using epidermis specific RNA-seq data with controls at all time points. Fields included are AGI, Alias(es), Cluster number, and a flag indicating whether the gene was differentially expressed at 6, 12, 18, 24, 30 or 36 hours. **Table 03.** List of 337 motifs identified using AME (65) with the DAP-seq (66) database. **Table 04.** List of 29 Consistent transcription factors identified in 20 runs of logistic regression with LASSO. Name, AGI and GO terms identified on arabidopsis.org (TAIR) are provided. **Table 05.** List of putative targets associated with each of the 29 consistent transcription factors. List includes the name of transcription factor, AGI of target, flag indicating whether target is 1) a known iron gene and/or 2) a transcription factor. **Table 06.** GO terms enriched in the group of targets associated with each of 29 consistent transcription factors. **Table 07.** Subset of GO terms from Supplementary Table 03 that include reference to "iron". GO ID, GO term, adjusted p-value and transcription factor name or AGI provided. **Table 08.** Subset of GO terms from Supplementary Table 03 that include reference to "root". GO ID, GO term, adjusted p-value and transcription factor name or AGI provided. **Table 09.** Subset of GO terms from Supplementary Table 03 that include reference to "epidermis". GO ID, GO term, adjusted p-value and transcription factor name or AGI provided.

## Data Availability

Raw sequence data (FASTQ files) and RPKM values have been submitted to NCBI GEO and are available for download (Accession # GSE228011). *Arabidopsis thaliana* pWER::GFP epidermis marker line (WEREWOLF; AT5G14750: Lee & Schiefelbein, 1999) in Columbia (Col-0) seeds were generated as described in Lee & Schiefelbein, 1999, and donated to, maintained by and purchased from Arabidopsis Biological Resource Center; stock number CS66493 and this material was used with permission.
