# Supplementary material for: Cellular clarity: a logistic regression approach to identify root epidermal regulators of iron deficiency response
Source: BMC Genomics. 2023 Oct 18;24:620. doi: 10.1186/s12864-023-09714-6 (PMC10583470; doi:10.1186/s12864-023-09714-6)
Supplement: Supplementary file 1 — Additional file 1: Figure. S01. Expression profiles of 2,739 DEGs. 50 clusters were identified using DPGP clustering algorithm. Cluster 25 and 26 were enriched in iron related GO terms. Figure. S02 GO Enrichment of putative targets for a subset of GO terms. Figure. S03. Expression values of 29 TFs identified using Logistic Regression. [file 12864_2023_9714_MOESM1_ESM.docx]

# Supplementary Data


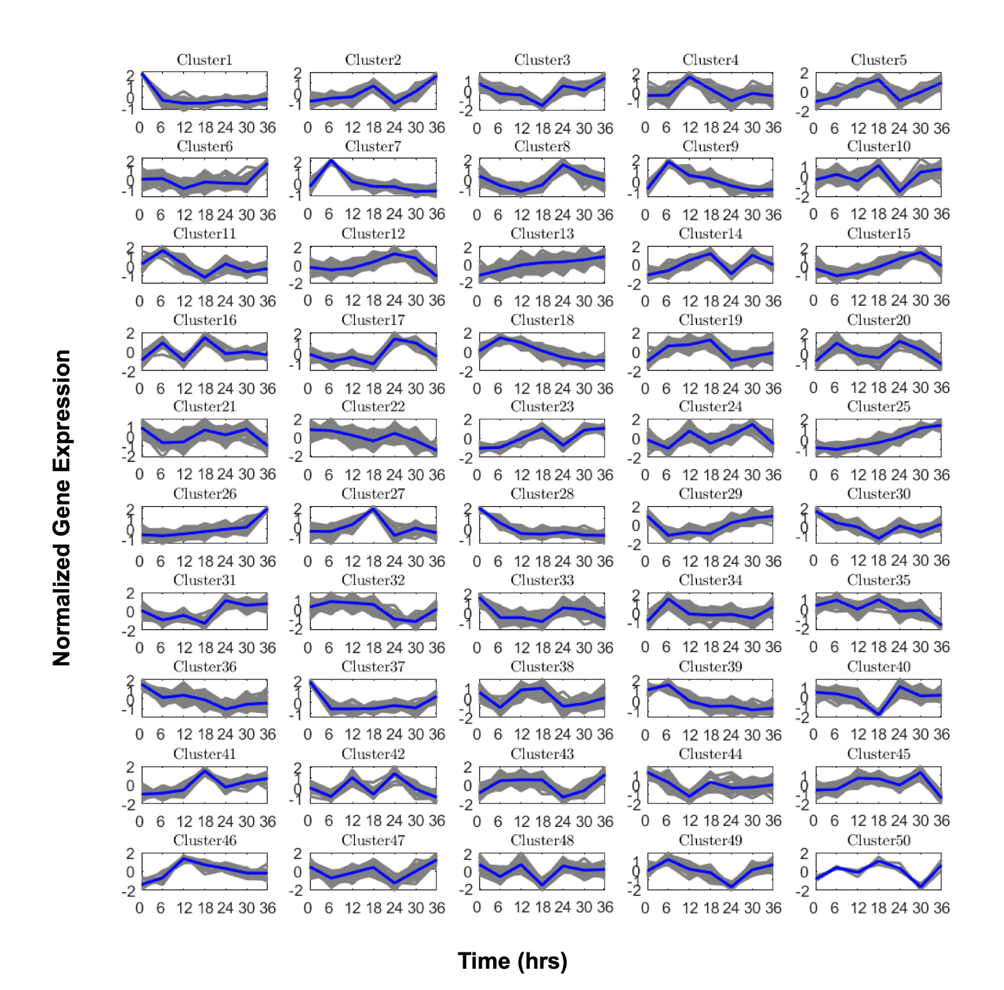


**Figure S01.** Expression profiles of 2,739 DEGs. 50 clusters were identified using DPGP clustering algorithm. Cluster 25 and 26 were enriched in iron related GO terms.


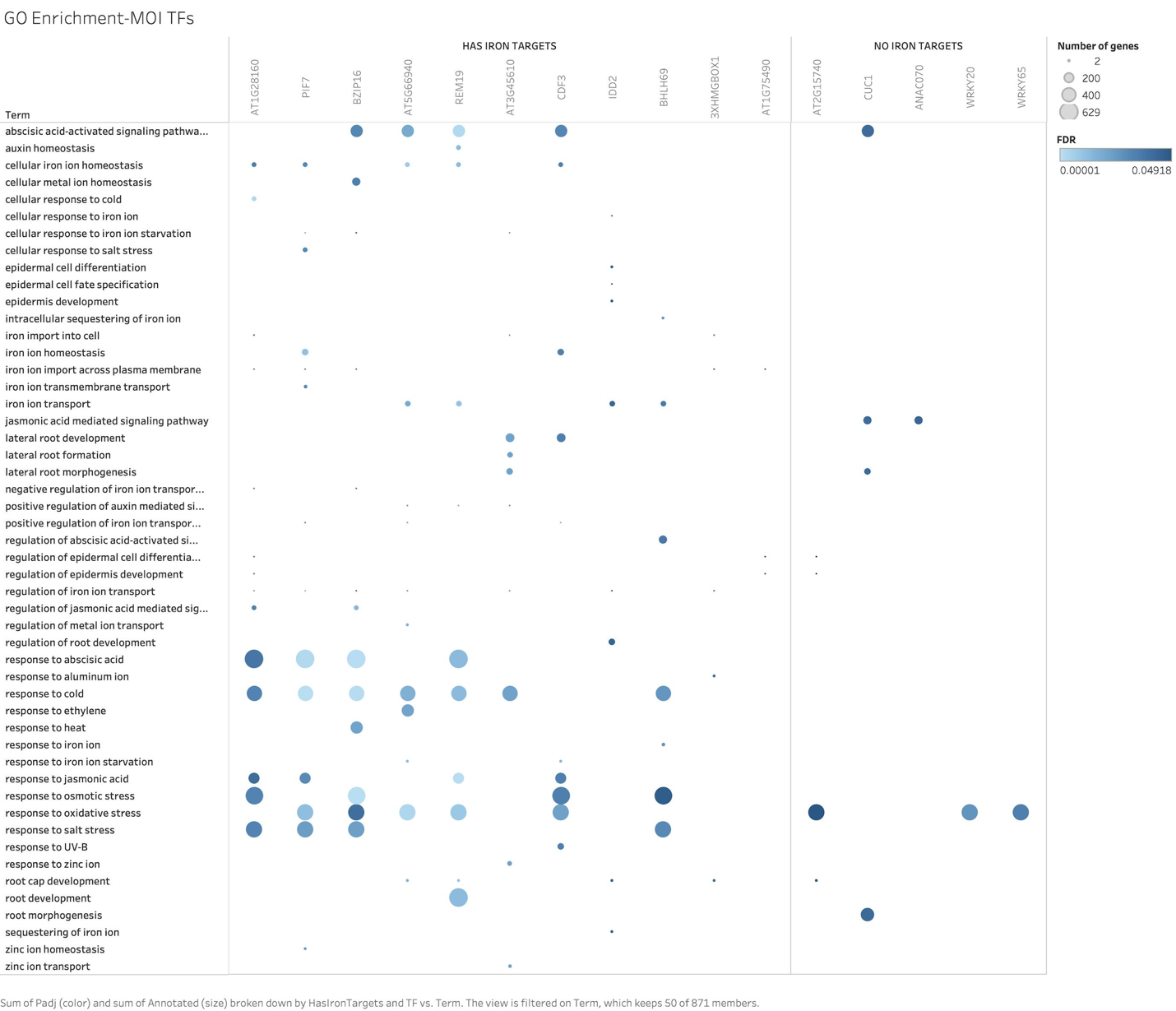


**Figure S02.** GO Enrichment of putative targets for a subset of GO terms. TFs included on this chart are a subset of 16 of the 23 TFs that had GO enriched terms in these categories. Size of circles is relative to number of genes enriched for the term while the color provides information about the FDR (darker color means higher adjusted p-value).


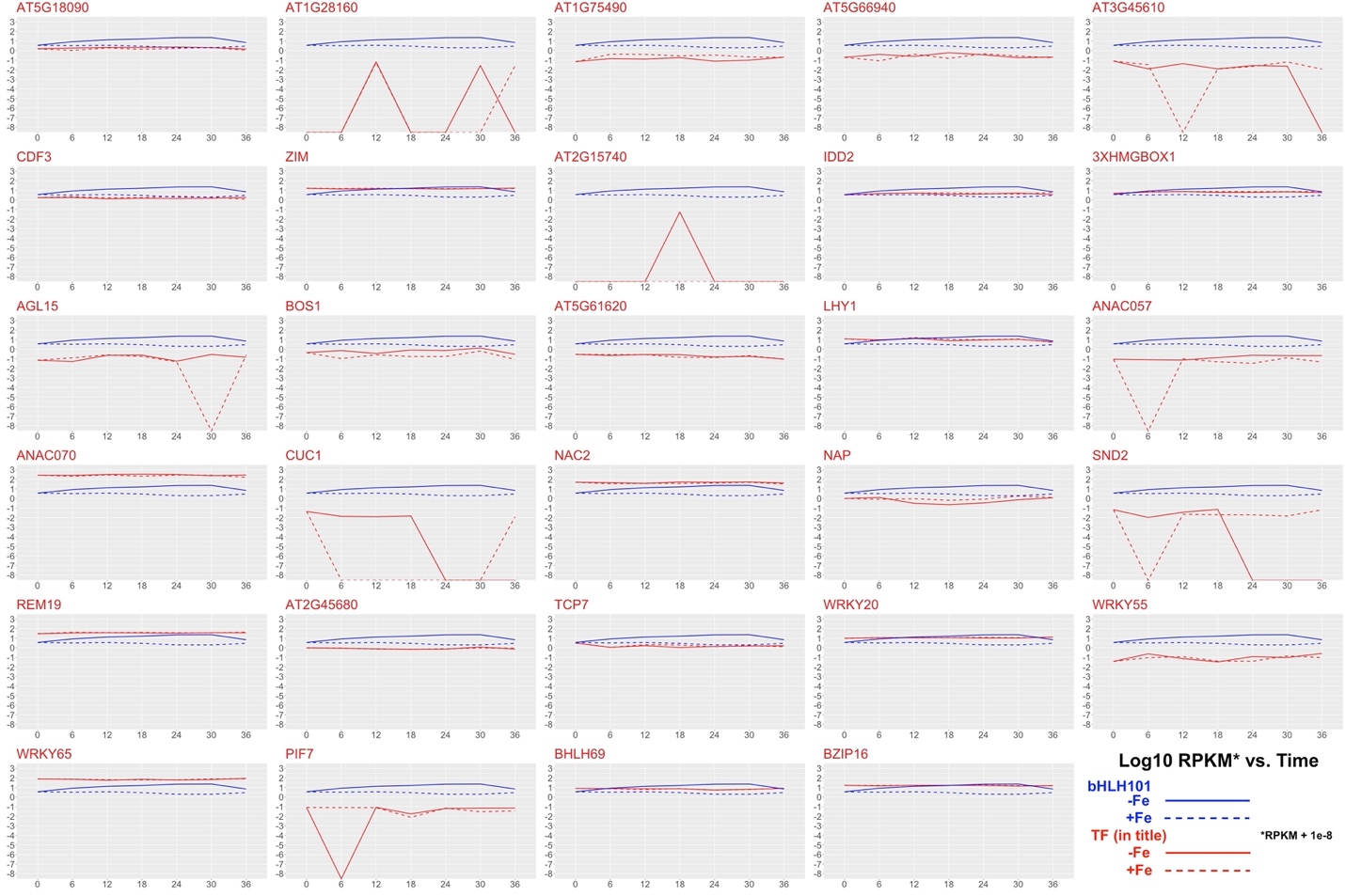


**Figure S03.** Expression values of 29 TFs identified using Logistic Regression. Log_10_ (RPKM+1e-8) values vs. time for 29 TFs (Red) are compared to bHLH101 (Blue), which is differentially expressed at all timepoints, under +Fe (dashed line) and -Fe (solid line). Of the 29 TFs only 1 (ANAC070) was found to be differentially expressed at any timepoint. The remaining genes are basally expressed, but not differentially expressed in response to -Fe. A small value, 1e-8, was added to the RPKM to deal with 68 measurements = 0 which leads to infinite Log_10_ values. The addition of this value does not substantively change the RPKM measurement for purposes of comparison.
